# The role of highly dispersed silica nanoparticles in the realization of the effects of granulosa on the maturation and fertilization competence of Sus scrofa domesticus oocytes

**DOI:** 10.18699/VJGB-22-30

**Published:** 2022-05

**Authors:** T.I. Kuzmina, I.V. Chistyakova, A.O. Prituzhalova, D.N. Tatarskaya

**Affiliations:** Russian Research Institute of Farm Animal Genetics and Breeding – Branch of the L.K. Ernst Federal Research Center for Animal Husbandry, Pushkin, St. Petersburg, Russia; Russian Research Institute of Farm Animal Genetics and Breeding – Branch of the L.K. Ernst Federal Research Center for Animal Husbandry, Pushkin, St. Petersburg, Russia; Russian Research Institute of Farm Animal Genetics and Breeding – Branch of the L.K. Ernst Federal Research Center for Animal Husbandry, Pushkin, St. Petersburg, Russia; Pushkin Leningrad State University, Pushkin, St. Peterburg, Russia

**Keywords:** porcine oocytes, maturation in vitro, highly dispersed silica nanoparticles, apoptosis, granulosa, ооциты свиней, созревание in vitro, наночастицы высокодисперсного кремнезема, апоптоз, гранулеза

## Abstract

Reproductive technologies are some of the key directions in the context of the need to preserve and select highly productive farmed animals in terms of economically useful traits. Improvements of the existing models of the in vitro oocyte maturation system help to solve the problem of low yield of porcine embryos at the final stages of preimplantation development. In the present study, a model of culture medium for gametes (NCSU-23 with 10 % homologous follicular fluid, 10 IU hCG and 10 IU eCG) modernized by the addition of 1·106 granulosa cells (GCs) per ml and 0.001 % of highly dispersed silica nanoparticles (HDSn) is proposed for use in the IVM and IVF technology of donor porcine oocytes. Analysis of the oocyte chromatin status by the Tarkowsky method and assessment of the level of destructive changes in chromatin (apoptosis, pyknosis) revealed a significant percentage increase in matured oocytes and a decrease in the proportion of granulosa cells with degenerated chromatin when using the original culture system. The positive effects of a joint addition of GCs and HDSn to the maturation system have made it possible to increase the indicators of the meiotic maturation and fertilization of oocytes. Optimal results of developmental competence of oocytes were achieved with the joint use of GCs and HDSn in the maturation system (the proportion of matured cells reached 89 %, the level of oocytes with chromosome degeneration was 12 %, 39 % of embryos reached the final stage of preimplantation development). The positive effect of HDSn on oocyte fertilization was accompanied by an abrupt decrease in destructive processes in GCs during culture in the presence of HDSn. The level of somatic cells with pyknotic nuclei was 32 % and the level of apoptosis (TUNELtest), 21 %, compared with the control (43 and 31 %, p <0.01, respectively). Thus, a high efficiency of the porcine oocyte maturation system in the joint culture of gametes with GCs and HDSn was revealed. It makes it possible to recommend a model of this culture medium at the IVM of female gametes of Sus scrofa domesticus for improving the quality of donor oocytes used in cell and genetic engineering.

## Abstract (ru)

Репродуктивные технологии являются одним из ключевых направлений в условиях необходимости сохранения и отбора выдающихся по хозяйственно полезным признакам особей сельскохозяйственных животных. Совершенствование имеющихся моделей созревания ооцитов in vitro в различных вариациях способствует решению проблемы низкого выхода эмбрионов свиней на завершающих стадиях доимплантационного развития. В настоящем исследовании с использованием технологии созревания и оплодотворения донорских ооцитов свиней in vitro предложена модель среды для культивирования гамет (NCSU-23 с 10 % гомологичной фолликулярной жидкостью, 10 МЕ ХГЧ и 10 МЕ ХГ лошади), модернизированная введением 1 · 106 клеток гранулезы (КГ) на 1 мл среды и 0.001 % наночастиц высокодисперсного кремнезема (нВДК). Анализ статуса хроматина ооцитов по методу Тарковского и оценка уровня деструктивных изменений хроматина соматических клеток овариальных фолликулов (апоптоз, пикноз) выявили значительное повышение показателей ядерного созревания гамет и снижение доли клеток гранулезы с дегенерированным хроматином при применении разработанной системы культивирования. Обнаружено позитивное влияние совместного введения КГ и нВДК в систему дозревания, позволившего увеличить показатели мейотического созревания и оплодотворяемости ооцитов. Оптимальные показатели фертильности ооцитов достигнуты при сочетанном использовании в системе дозревания КГ и нВДК (доля созревших клеток достигла 89 %, уровень ооцитов с дегенерацией хромосом составил 12 %, 39 % эмбрионов достигли завершающей стадии доимплантационного развития). Положительный эффект нВДК на показатели оплодотворяемости ооцитов сопровождался резким снижением деструктивных процессов в КГ при их культивировании в присутствии нВДК. Уровень пикнозов составил 32 %, а уровень апоптозов (TUNEL-test) – 21 % по сравнению с контролем (43 и 31 % соответственно, р <0.01). Таким образом, выявлена высокая эффективность системы созревания ооцитов свиней в условиях совместного кокультивирования гамет с КГ и нВДК, что позволяет рекомендовать модель разработанной среды в технологии экстракорпорального созревания женских гамет Sus scrofa domesticus для повышения качества донорских яйцеклеток, используемых в клеточной и генетической инженерии.

## Introduction

Cell reproductive and DNA biotechnologies play an important role in the intensification of breeding process in animal husbandry because they are a tool for increasing the number of individuals outstanding in economic traits (Romar et al., 2019). Biotechnological interest in the species Sus scrofa domesticus has increased because it can be used in biomedicine, due to the features of its physiology (proximity to the species Homo sapiens), for organ xenotransplantation. In vitro production of viable native and reconstructed (cloned, transgenic) porcine embryos on a mass scale is possible; however, now, certain steps in the technology of in vitro maturation of S. scrofa domesticus eggs and their fertilization require improvement (Fowler et al., 2018). Development of standardized protocols for methodology of obtaining porcine embryos in vitro is necessary to take full advantage of the possibilities of innovative cellular reproductive technologies in porcine breeding and biomedicine, including production of genetically modified pigs.

Effectiveness on various stages of extracorporeal production of porcine embryos is ambiguous. Improvement of oocyte maturation systems, low percentage of monospermic zygotes and zygotes that develop to the final stage of preimplantation development (blastocyst) require solutions (Martinez et al., 2019). Nowadays, there are many works on the development of a unified maturation system of donor porcine oocytes in vitro, but yield of embryos at the final stages of preimplantation development still does not exceed 45–50 % (Soriano-Úbeda et al., 2017).

The abovementioned allows us to define the task of modeling the culture media composition for completion of porcine oocyte meiotic maturation in vitro as highly relevant. In vivo, an egg is forming in close relationship with the somatic cells of the ovarian follicle (cumulus, granulosa), which produce a number of bioactive molecules involved in the growth and maturation of oocytes. The pioneering works of L.R. Abeydeera showed effectiveness of using follicle walls and follicular fluid as part of oocyte maturation systems (Abeydeera et al., 1998). However, procedures to dissect follicle, objectivity of its quality evaluation by embryotechnologist prolong the duration of the first stage of embryo production technology. The use of innovative materials, including their nanoscale particles, in in vitro maturation system of animal gametes is a rapidly developing branch of bionanotechnology (Remião et al., 2018). Many researchers have evaluated the cyto- and gene-toxicity of nanoparticles of different origin on mammalian germ cells (Roy et al., 2020).

Our previous studies revealed positive effects of HDSn on cell compartments functioning of native and devitrified female gametes of farm animals, destructive chromatin processes in the nuclei of germinal and somatic cells of ovarian follicles (Kuzmina et al., 2017, 2020). Based on these considerations, it seems logical to add granulosa cells into the basic culture media as a potential supplier of natural origin biologically active substances, primarily steroids, and nanoparticles of different origin.

The aim of this study was to evaluate the role of highly dispersed silica nanoparticles in realizing the effects of the addition of granulosa cells into the system of extracorporeal maturation of porcine oocytes on gamete fertility indices.

## Materials and methods

All reagents used in the experiments, except as indicated in the text, were produced by Sigma-Aldrich (USA). Plastic laboratory glassware was from BD Falcon™ (USA).

In experiments we used cumulus oocyte complexes (COC) isolated from the antral follicles of post mortem ovaries of S. scrofa domesticus landrace breed at the age of 6–8 months. The ovaries after ovariectomy of animals at a local slaughterhouse were delivered to laboratory in 0.9 % NaCl solution at 30–35 °C containing 100 IU/ml penicillin, 100 μg/ml streptomycin, and 0.25 ng/ml amphotericin. COCs were aspirated from antral follicles (with high turgor, from 3 to 6 mm diameter, and a high degree of vascularization). Oocytes with homogeneous ooplasm, zone pellucid uniform in width, surrounded by a compact layer of cumulus cells (at least 5–6 layers) were used in experiments.

After morphological evaluation, 40–50 COCs were placed in droplets (500 μl volume) of culture media with the following composition: Group I – synthetic culture medium North Carolina State University-23 (NCSU-23) + 10 IU human chorionic gonadotropin + 10 IU equine chorionic gonadotropin + 10 % follicular fluid (follicle diameter 3–6 mm); Group II – synthetic culture medium NCSU-23 + 10 IU human chorionic gonadotropin + 10 IU equine chorionic gonadotropin + 10 % follicular fluid (follicle diameter 3–6 mm) + 0.001 % HDSn; Group III – synthetic culture medium NCSU-23 + 10 IU human chorionic gonadotropin + 10 IU equine chorionic gonadotropin + 10 % follicular fluid (follicle diameter 3–6 mm) + 1·106 granulosa cells (GC) per ml of medium; Group IV – synthetic culture medium NCSU-23 + 10 IU human chorionic gonadotropin + 10 IU equine chorionic gonadotropin + 10 % follicular fluid (follicle diameter 3–6 mm) + 1·106 GC per ml of medium + 0.001 % HDSn. HDSn were synthesized in the Chuiko Institute of Surface Chemistry, National Academy of Sciences of Ukraine. Concentration was chosen according to the recommendations of developers (Zyuzyn et al., 2015). COCs were cultured for 22 hours at 38.5 °C in the atmosphere of 5 % CO2 in aforementioned media, then the media were changed, hormones were excluded in all studied groups and they were cultured for the next 22 hours.

The chromatin status of oocytes at meiotic maturation and the level of pyknosis in granulosa cells were tested with cytology method (Kuzmina et al., 2008). Oocytes were placed for 5–10 min in a warm (37 °C) 0.9 % hypotonic solution of 3-substituted sodium citrate and purified from cumulus. Then cells were transferred on dry non-fat glass and fixed with a mixture of methanol and acetic acid (3:1). Dried samples of oocyte and granulosa cells were stained with 4 % Romanovsky–Giemsa solution (azure-eosin) for 3–4 min.

The level of apoptosis in GCs after culture for 22 hours in NCSU-23 medium with 10 IU human chorionic gonadotropin, 10 IU equine chorionic gonadotropin, 10 % follicular fluid (follicle diameter 3–6 mm) and 22 hours later (total culture time 44 hours) after culture medium change (exclusion of hormonal supplements) was assessed by TUNEL (Janowski et al., 2012). The experimental group was supplemented with 0.001 % HDSn at all stages of culture.

We used modified mTBM medium containing 113.1 mM NaCl, 3.0 mM KCl, 7.5 mM CaCl2·2H2O, 20.0 mM Tris, 11.0 mM glucose, 5.0 mM sodium pyruvate, 1 mM caffeine and 0.1 % BSA for in vitro fertilization, after 44 hours of culture, the oocytes were mechanically (by pipetting) released from the cumulus cells. Then in amount of 10 pcs. placed in drops of mTBM medium (volume 90 μl under paraffin oil) in 35 mm culture dishes for 30 min in CO2 incubator for equilibration. The oocytes were fertilized with native sperm (initial concentration in diluent 3·109 spermatozoa per ml). After 3-fold centrifugation (80 g for 3 min at room temperature), 10 ml of sperm suspension was resuspended in 10 ml of DPBS with 0.1 % BSA and sperm concentration was adjusted to 2·106 cells per ml. 16 μl of sperm suspension was added to 90 μl droplets with oocyte and cultured in a CO2 incubator at 38.5 °C in an atmosphere of 5 % CO2 and 90 % humidity. After 6 hours of incubation with spermatozoa, the oocytes were transferred to 500 μl of NCSU-23 medium with 0.4 % BSA for culturing in a CO2 incubator for 7 days at 38.5 °C in an atmosphere of 5 % O2, 5 % CO2 and 90 % N2 with medium changes every 48 hours of culture (Egerszegi et al., 2010).

To determine the level of apoptosis in GCs, its suspension was placed on poly-L-lysine-coated slides and dried. Next, apoptosis levels were tested according to the manufacturer’s instructions and the method adapted for granulosa cells presented by Janowski et al. (2012). For this purpose, GCs were fixed in 4 % (v/v) paraformaldehyde solution for 30 min, incubated for 2 min in 10 % Triton X-100 solution on 0.1 % sodium citrate. Then GCs were incubated with TUNEL reagent (Roche Diagnostics, GmbH, Mannheim, Germany) for 60 min at 37 °C in the dark. After incubation cells were washed in DPBS solution, stained in 0.1 % (w/v) propidium iodide solution (20 min exposure), washed again in DPBS, and exposed for 1 hour in the dark at room temperature. Samples were stored in the refrigerator at +3 to +5 °C. Samples were analyzed using a ZEISS AxioLab. fluorescence microscope A1 (Carl Zeiss, Germany).

The results were processed using the SigmaStat statistical software package (Jandel Scientific Software, USA). Pearson’s χ2 test was used to assess the reliability of frequency variables. Significance of differences between the compared values was assessed at the following levels: p < 0.05, p < 0.01, and p < 0.001 for 3–5 independent experiments.

## Results and discussion

**Fig. 1.**
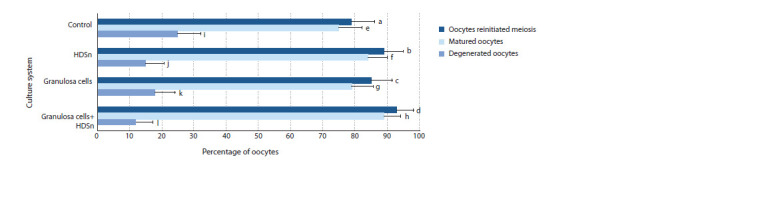
Indicators of porcine oocytes chromatin status after culture with granulosa cells and HDSn (time of culture – 44 hours, number of oocytes – 600). * Differences are statistically significant (χ2 test): a : b; c : d; e : f; g : h; i : j – р ˂ 0.05; a : d; e : h; i : l – р ˂ 0.1.

Granulosa and cumulus cells produce a great number of growth and other factors determining oocyte formation and subsequent embryo development (Canipari, 2000). Nanoparticles of various chemical compounds, including HDSn, can synchronize the nuclear and cytoplasmic maturation of animal oocytes and protect intracellular components from factors detrimental to their functioning, including reactive oxygen species (ROS) (Kuzmina et al., 2017, 2020). Data of chromatin status analysis in porcine oocytes were cultured with granulosa cells and HDSn are presented in Figure 1.

**Fig. 2.**
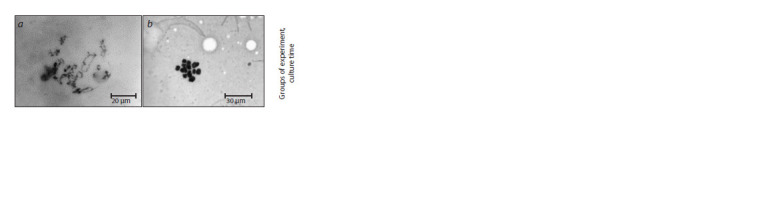
Representative image of S. scrofa domesticus oocytes chromatin at diplotene (a) and metaphase II (b) stages. Cytological sample, staining with azure-eosin according to Romanovsky– Giemsa, microscope ZEISS AxioLab. A1, Carl Zeiss.

Addition of HDSn to culture medium promoted re-initiation and completion of meiosis (Fig. 2) in oocytes cultured without GC compared to cells in the control group (79 and 75 % versus 89 and 84 %, p < 0.05). Moreover, the stimulating effect of HDSn’s on oocyte maturation was also observed in co-cultured gametes with GC (85 and 79 % versus 93 and 89 %, p < 0.05). It is important to note that adding HDSn resulted in a decrease in the percentage of degenerated oocytes in experimental groups compared to control groups cultured with or without somatic cells (15 and 12 % versus 25 and 18 %, p < 0.01).

**Fig. 3.**
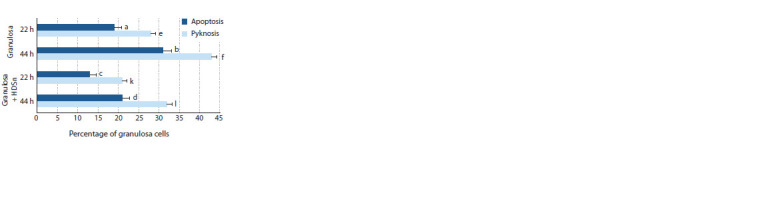
Destructive processes of chromatin in granulosa cells of porcine ovarian follicles (number of cells –7539). * Differences are statistically significant (χ2 test): a : c; a : d; e : l – p ˂ 0.05; a : b; b : c; b : d; c : d; e : f; e : k; f : k; f : l; k : l – p ˂ 0.01.

In the second series of experiments, we evaluated the effect of HDSn on destructive processes in GCs during in vitro culture (Fig. 3). The inhibitory effect of HDSn on destructive processes in chromatin (apoptosis, pyknosis) of granulosa cells during prolonged culture was shown. Thus, after 22 hours of culture, proportion of cells with pyknotic nuclei was lower by 7 % in the group cultured with HDSn compared to the control (21 and 28 %, p < 0.01), and proportion of apoptotic cells – by 6 % (13 and 19 %, p < 0.05). After 44 hours of culture, proportion of cells in pyknotic state reached 43 % (p < 0.01) in the control group and the level of apoptotic cells reached 31 % (p < 0.01). In the experimental group, these indices were significantly lower (32 and 21 %, p < 0.01).

**Fig. 4.**
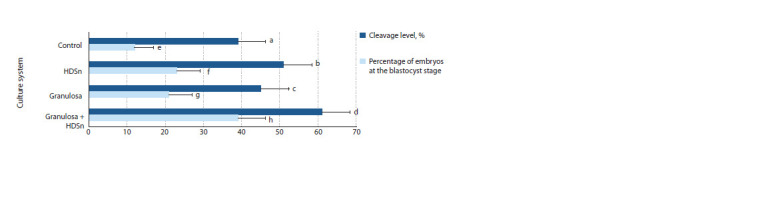
Analysis of fertility indicators of S. scrofa domesticus oocytes matured in different culture systems (number of oocytes – 736). * Differences are statistically significant (χ2 test): a : b, e : g – p ˂ 0.05; a : d, c : d, e : f, e : h, f : h, g : h – p ˂ 0.01.

The results of analysis fertility parameters of oocytes matured in various systems are shown in Fig. 4. Fertilizable addition of HDSn to maturation medium provided an increase in the level of fertilized oocytes, which was expressed by an increase of 12 % in the cleavage level (51 %, p < 0.05) and 11 % in the yield of blastocysts (23 %, p < 0.01) and an increase in the yield of preimplantation embryos at blastocyst stage compared with the control group (39 and 12 %, respectively). At the same time, maximum fertilization rates were observed in the group of oocytes co-cultured with granulosa and HDSn cells (61 and 39 %, respectively, p <0.01 versus control groups).

Oxidative stress is one of the main factors reducing development competence of oocytes at culture (Wei et al., 2016). Positive effect of nanoparticles on oocyte maturation can probably be explained by the ability of HDSn to level the damaging effect of free-radical processes at cell culture by reducing the formation of oxidative modification products of proteins (Savchenko, 2013). In addition, as a result of oxidative stress on cells in the endoplasmic reticulum (ER), occur synthesis and assembly of lipid droplets (LD), which act as a protective mechanism when reactive oxygen species act on the membrane structures of organelles, as well as supply mitochondria with fatty acids for ATP production (Lee et al., 2012; Zhang X., Zhang K., 2012). It is known that the intracellular form of LDs in the shape of “granules” and Fig. 1. Indicators of porcine oocytes chromatin status after culture with granulosa cells and HDSn (time of culture – 44 hours, number of oocytes – 600). * Differences are statistically significant (χ2 test): a : b; c : d; e : f; g : h; i : j – р ˂ 0.05; a : d; e : h; i : l – р ˂ 0.1. Fig. 2. Representative image of S. scrofa domesticus oocytes chromatin at diplotene (a) and metaphase II (b) stages. Cytological sample, staining with azure-eosin according to Romanovsky– Giemsa, microscope ZEISS AxioLab. A1, Carl Zeiss. Fig. 3. Destructive processes of chromatin in granulosa cells of porcine ovarian follicles (number of cells –7539). * Differences are statistically significant (χ2 test): a : c; a : d; e : l – p ˂ 0.05; a : b; b : c; b : d; c : d; e : f; e : k; f : k; f : l; k : l – p ˂ 0.01. their diffuse arrangement provide fatty acid mobilization, determining normal maturation of cumulus oocyte complexes (Bradley et al., 2019). It was shown that the addition of HDSn to culture medium provides an increase in the level of oocytes with the LDs with diffuse localization, which, as indicated earlier, ensures normal gamete development (Novichkova, Kuzmina, 2019).

It is known that communication of GCs, as well as interaction of cumulus cells with the oocyte, determines the growth and formation of female gamete. Successful oocyte maturation and further embryonic development depend on the action of certain hormones, in particular progesterone and estradiol secreted by granulosa cells. In turn, the effect of these hormones on oocytes is mediated by the radial crown cells expressing FSHR (follicle-stimulating hormone receptor), which is necessary for cumulus cell proliferation and normal gamete development (Okazaki et al., 2003). It was shown that the addition of progesterone and β-estradiol in culture medium increases the level of FSHR expression, cumulus cell survival, and reduces the level of apoptosis (Okamoto et al., 2016). HDSn prevent apoptosis in somatic cells and male gametes of animals by stimulating antioxidant system through interacting with receptors on the cell surface (Boytseva et al., 2017; Kuzmina et al., 2017).

## Conclusion

The development of an effective protocol for obtaining native and reconstructed S. scrofa domesticus embryos in vitro will significantly intensify stages of innovative cellular reproductive technologies used in animal husbandry, veterinary medicine and biomedicine. The aims of the present study are to improve extracorporeal maturation system of donor porcine oocytes to obtain oocytes competent for fertilization and embryo development. Considering the importance of somatic cells of ovarian follicles in the formation of a mature oocyte, coculture of cumulus-oocyte complexes with granulosa cells was used in the experiments. Maturation system was upgraded by the addition of HDSn into culture medium.

Experiments revealed a positive effect of the developed system on indicators of fertility of oocytes (yield of matured oocytes, cleavage, and level of embryos that reached the final stage of preimplantation development). The most positive effect was observed when HDSn and granulosa cells were used in the culture system together. High fertility rates of oocytes matured in medium with HDSn are probably explained by a reduced level of destructive changes in surrounding cumulus cells (subpopulation of granulosa cells).

The study found that the addition of HDSn into the culture medium causes the levels of apoptosis and pyknosis in granulosa cells to decrease, which indicates an increase in the number of viable cells, their hormonesynthetic activity and provides physiological processes involved in formation of oocytes with high fertility. The most significant indicator in evaluation of the effectiveness of any culture system for maturation of oocytes is the embryo yield. In our studies, the yield of embryos at the final stage of preimplantation development was the highest (39 %) in the case of the joint use of HDSn and granulosa cells in oocyte maturation system. Results of the study allow to recommend the developed culture system for extracorporeal maturation of donor oocytes of S. scrofa domesticus.

## Conflict of interest

The authors declare no conflict of interest.
